# Monthly measurement of child lengths between 6 and 27 months of age in Burkina Faso reveals both chronic and episodic growth faltering

**DOI:** 10.1093/ajcn/nqab309

**Published:** 2021-10-12

**Authors:** Ilana R Cliffer, William A Masters, Nandita Perumal, Elena N Naumova, Augustin N Zeba, Franck Garanet, Beatrice L Rogers

**Affiliations:** Friedman School of Nutrition Science and Policy, Tufts University, Boston, MA, USA; Global Health and Population Department, Harvard TH Chan School of Public Health, Harvard University, Boston, MA, USA; Friedman School of Nutrition Science and Policy, Tufts University, Boston, MA, USA; Global Health and Population Department, Harvard TH Chan School of Public Health, Harvard University, Boston, MA, USA; Friedman School of Nutrition Science and Policy, Tufts University, Boston, MA, USA; Department of Nutrition, Health Sciences Research Institute, National Center for Scientific and Technological Research (Institut de Recherche en Sciences de la Santé, Centre National de la Recherche Scientifique et Technologique), Ouagadougou, Burkina Faso; Department of Nutrition, Health Sciences Research Institute, National Center for Scientific and Technological Research (Institut de Recherche en Sciences de la Santé, Centre National de la Recherche Scientifique et Technologique), Ouagadougou, Burkina Faso; Friedman School of Nutrition Science and Policy, Tufts University, Boston, MA, USA

**Keywords:** nutrition, child growth, anthropometry, undernutrition, linear growth

## Abstract

**Background:**

Linear growth faltering is determined primarily by attained heights in infancy, but available data consist mainly of cross-sectional heights at each age.

**Objectives:**

This study used longitudinal data to test whether faltering occurs episodically in a few months of very low growth, which could potentially be prevented by timely intervention, or is a chronic condition with slower growth in every month of infancy and early childhood.

**Methods:**

Using anthropometric data collected monthly between August 2014 and December 2016, we investigated individual growth curves of 5039 children ages 6–27 mo in Burkina Faso (108,580 observations). We evaluated growth-curve smoothness by level of attained length at ∼27 mo by analyzing variation in changes in monthly growth rates and using 2-stage regressions: *1*) regressing each child's length on their age and extracting *R*^2^ to represent curve smoothness, initial length, and average velocity by age; and *2*) regressing extracted parameters on individual-level attained length.

**Results:**

Short children started smaller and remained on their initial trajectories, continuously growing slower than taller children. Growth between 9 and 11 mo was the most influential on attained length; for each 1-cm/mo increase in growth velocity during this period, attained length increased by 6.71 cm (95% CI: 6.59, 6.83 cm). Furthermore, a 0.01 increase in *R*^2^ from individual regression of length on age was associated with a 3.10-cm higher attained length (95% CI: 2.80, 3.41 cm), and having 2 consecutive months of slow growth (<15^th^ centile relative to the sample) was associated with 1.7-cm lower attained length (95% CI: −1.80, −1.59 cm), with larger effects in younger children, suggesting that smoother growth patterns were also associated with higher attained length.

**Conclusions:**

Children who experience extreme growth faltering are likely less resilient to systematic growth-limiting conditions as well as episodic insults to their growth.

This trial was registered at clinicaltrials.gov as NCT02071563.

## Introduction

Linear growth, measured by a child's length or height, is an important measure of overall child health and their potential for thriving physically, cognitively, and economically throughout life (1, 2). Slower than expected growth compared to a standard reference population (3, 4) is referred to as linear growth faltering. Typically, linear growth faltering is measured by declines in height:age ratios relative to the WHO standards (1, 5). These standards reflect child growth in ideal environments with no constraints for growth, as defined by high socioeconomic status and optimal feeding practices among children from 5 different regions of the world (5). Whereas linear growth faltering is not common in high-income countries where more children live in such ideal environments, it remains a prevalent issue in low- and middle-income countries despite substantial investments in interventions to support early childhood growth (6).

Understanding the etiology of growth faltering onset and the sustained intensity of suboptimal growth (7) is critical to designing interventions and policies that effectively reduce the burden of growth faltering. Current knowledge of the timing of growth faltering is largely informed by cross-sectional studies that pool data across separate cohorts of children in different age ranges, which fails to identify onset and patterns of growth faltering among individuals within populations (3, 8–10). A landmark study by Victora et al. (8) in 2010 used cross-sectional data from 54 countries to identify the critical window of opportunity for child growth in the first 1000 d of life, from early pregnancy to 2 y, but left important questions unanswered. Although we know that more children experience growth faltering in the first 2 y of life, few studies have collected repeated measures on the same children at a high enough frequency to assess when and how individual children experience their growth faltering during this period. Cross-sectional data are inadequate to assess growth of individuals, because one's growth trajectory is determined by current height or weight conditional on previous measurements (11). The normal growth process was suggested to be saltatory, with extended periods of stasis punctuated by short phases of growth (12), although consensus from researchers in the field was not reached (13). The extent to which this process occurs similarly among children with suboptimal growth has not been examined in depth.

We used longitudinal data from a cohort of ∼5000 Burkinabè children measured monthly for an average of 21 mo each, at the ages of 6–27 mo, to determine the timing and pattern of growth faltering in individuals. We defined human growth as the change in the size of body measurements between 2 subsequent ages (14), and growth faltering by level of attained length at study end (∼27 mo of age). Using a combination of data visualizations, individual regressions of length on postnatal age for each child in our sample, and analysis of the frequency and duration of slow growth episodes defined by length velocity cutoffs relative to others in the sample, we identified influential growth periods as children age and evaluated whether growth faltering happens through continuous or intermittent slow growth.

## Methods

### Study design and data source

This is a secondary analysis using data collected during a blanket supplementary feeding trial (NCT02071563) comparing the cost-effectiveness of 4 foods in the prevention of stunting and wasting in children 6–23 mo old in Sanmatenga Province, Burkina Faso. Between August 2014 and December 2016, children whose caregivers lived in Sanmatenga Province and had been receiving a supplementary food (∼500 kcal/d of either a fortified blended flour or a lipid-based nutrient supplement, delivered monthly) starting during pregnancy, as part of the feeding program, were enrolled in the study when they reached 6 mo of age and measured monthly (recumbent length, weight, midupper arm circumference) for 18 mo while receiving foods and for 3 consecutive months postintervention. A total of 6112 children were measured a mean of 21 times each, adding up to 129,944 observations over ∼2.5 y. Further details of the original study are explained elsewhere (15).

Exclusion criteria for the original intervention were severe acute malnutrition (children with a midupper arm circumference < 11.5 cm were referred to a health center) and age > 12 mo, although enrolling children >6 mo old was rare. Eligible children in the intervention zone were enrolled on a rolling basis until the sample size was reached after 10 mo. Intervention arms were geographically clustered; data were pooled and analyzed with statistical controls for study arm.

### Anthropometric measures and indexes

Anthropometric data were collected by trained enumerators who participated in standardization exercises every 3 mo. Measurements were done in duplicate for quality control, recorded on paper forms, and double-entered into a CSPro database and checked for consistency (16). During measurement, enumerators were instructed by a supervisor to perform a third measurement if the difference between the 2 measurements exceeded 0.2 cm, and the supervisor recorded the 2 closer measurements. Procedures for identification of implausible anthropometric values and data preparation are described in the supplementary data (**Supplemental Methods 1**). The final data set consisted of 5039 children between 6 and 27 mo old (82% of the original sample) who each had ≥20 repeated measurements (108,580 total observations). **Supplemental Figure 1** shows a participant flowchart showing how the final data set was derived. A sensitivity analyses data set with similar distributions of key variables to the full data set consisted of only complete cases with 22 measurements each (1158 children, 25,476 observations) (**Supplemental Table 1**).

Age- and sex-standardized length-for-age *z* scores (LAZs) were calculated using the WHO Child Growth Standards macro for Stata (5, 17), and length-for-age differences (LADs) or height-for-age differences were calculated manually in Stata using WHO growth reference tabulated median length or height values (5). Length velocity (cm/mo) was calculated by subtracting each month's length measurement from the previous measurement, dividing by the time gap between measurements, and multiplying by 30.44. The primary outcome of attained length was defined as the child's absolute length at the end of the study period (∼27 mo of age), in centimeters. We used attained length to improve on the binary population-based metric for stunting that has a biologically arbitrary cutoff <−2 SD based on the WHO child growth standards (4).

### Analytic methods

#### Growth-curve smoothness as a predictor of attained length

To assess the episodic compared with continuous nature of growth faltering, we examined whether growth-curve smoothness is an important predictor of attained length using 2-stage regression models. First, we regressed each individual child's length on their age, totaling 5039 separate regression models. We extracted model fit parameters from these models, including the *R*^2^ and *F*-statistics as measures of curve fit and smoothness, constant values as a measure of initial length at 6 mo, and age-term coefficients as a measure of average velocity. Second, we regressed the extracted parameters on individual-level attained length at study end. We hypothesized that if growth faltering occurs intermittently, a child will have more variance along their growth curve, and therefore a lower *R*^2^ from simple regressions of their length on their age. Thus, higher values of *R*^2^ indicate smoother growth, in which more of the variance in length is explained by age alone. It also follows that if higher attained length is the result of uninhibited growth spurts, a low *R*^2^ could also be associated with higher attained length.

To determine the appropriate functional form of the first-stage regressions, we compared Akaike's Information Criterion and the Bayesian Information Criterion between a linear regression model with a cubic term for age, linear splines with 6 knots evenly spaced, linear splines with 4 knots, and cubic splines. Linear splines with 6 knots had the best fit for the data. Individual regressions for each child, *i*, thus took the form:

(1) \begin{eqnarray*} {\rm{Lengt}}{{\rm{h}}_i} = {\beta _{0i}} + {\beta _{1i}}{\rm{age}} + \mathop \sum \limits_{k = 1}^K {b_k}\left( {{\rm{age}} - {\xi _k}} \right) + \end{eqnarray*}

where *K* is the number of knots, (age − *ξ_k_*)+ refers to the *k*^th^ linear function with a knot at *ξ_k_*, and

(2) \begin{eqnarray*} \left( {{\rm{age}} - {\xi _k}} \right) + = \left\{ {\begin{array}{@{}*{2}{c}@{}} {{\rm{age}} - {\xi _k}:}&{{\rm{if\ age}} - {\xi _k} > 0}\\ {0:}&{{\rm{if\ age}} - {\xi _k} \le 0} \end{array}} \right\} \end{eqnarray*}

We placed 6 knots at 9, 12, 15, 18, 21, and 24 mo; the slope of the relation between length and age changes at each knot based on the weight of each linear function,$\ {b_k}$. Although we imposed a common functional form on all individuals’ growth models, functional form may differ by child. We therefore performed sensitivity analyses using the candidate functional forms (**Supplemental Table 2**). Because these are individual regressions of length on age, overfitting is not an issue.

The second round of regressions to estimate the average association between model parameters from the first stage and attained length took the form:

(3) \begin{eqnarray*} {\rm{AttainedLengt}}{{\rm{h}}_i} &=& {\delta _{0i}} + {\delta _{1i}}\left( {{R^2}} \right) + {\delta _{2i}}\left( {{\rm{Intercept}}} \right)\\ && +\, {\delta _{wi}}\left( {\mathop \sum \limits_{k = 1}^K {b_k}\left( {{\rm{age}} - {\xi _k}} \right) + } \right) + {\varepsilon _i} \end{eqnarray*}

where for each child, *i, δ_w_* is the coefficient for each age spline: 6–8 mo, 9–11 mo, 12–14 mo, 15–17 mo, 18–20 mo, 21–23 mo, and 24–28 mo, the Intercept represents initial length, and *R*^2^ represents the smoothness of growth from the individual model.

To check that our inferences about the biological nature of growth faltering were made irrespective of original study arm, we checked for differences in mean *R*^2^ by attained length quintile, stratified by study arm. In all study arms, the differences in *R*^2^ from each quintile to the next were similar (**Supplemental Table 3**). We can therefore interpret our findings with the assumption that provision of different types of food supplements did not influence growth trajectories over time.

#### Variation of changes in monthly growth rates

To identify periods of slow growth, we identified all months in which a child grew more slowly than others of their age in our sample. The appropriate threshold for slow growth depends on the magnitude of measurement error relative to the prevalence of actual illness or inadequate diet. In this exploratory analysis, we used the lowest cutoff at which >95% of the children in the sample had ≥1 such month, to include all children who might have periods of slow growth. The threshold, set here based on length growth (cm/mo), was determined as growth <15^th^ centile for children of the same age. We tested how the frequency and duration of episodes of slow growth related to the child's attained length by the end of the study. Whereas a single month of slow growth could be caused by measurement error, randomness, or trend reversion, consecutive months of slow growth are likely to indicate an episode of illness or inadequate diet. Our primary hypothesis is that children who experience ≥2 consecutive months of slow growth have lower attained length by the end of the study, and that this effect is larger when slow growth occurs in younger children. The deficiency of models that use this threshold is that they do not consider the serial autocorrelation of the growth process. As such, we conducted sensitivity analyses using a second threshold of 2 consecutive negative residuals from regressions of length on age, conditional on initial length.

Visualizations and analyses were done in R Studio version 1.2.5033(18) and Stata version 16.1 (19).

### Ethics

Original data collection was approved by the Tufts University Health Sciences Institutional Review Board (IRB) (10899) and the ethics board of the Ministry of Health in Burkina Faso (2013-10-090). Secondary analysis for this article was deemed exempt by the Tufts University Health Sciences IRB (STUDY00000255) and was exploratory, with no prespecified endpoint.

## Results

### Sample anthropometric characteristics

**Table 1** shows a summary of anthropometric characteristics by quintile of attained length. Age at both first and last measurements was similar across quintiles; as such, attained length quintile was an appropriate indicator of growth faltering. There was a 5.3-cm difference in absolute length at first measurement between the highest and lowest quintiles, which increased to 9.1 cm at last measurement. LADs all decreased between the first and last measurements, regardless of quintile, but the decrease was larger in children in the lowest quintile of attained length.

**TABLE 1:** Sample anthropometric characteristics by quintile of attained length, main data set^1^

	Overall (*n* = 5039)	Quintile 1 (*n* = 1028)	Quintile 2 (*n* = 999)	Quintile 3 (*n* = 1010)	Quintile 4 (*n* = 1009)	Quintile 5 (*n* = 993)
Total observations, *n*	108,580	21,996	21,489	21,784	21,825	21,486
Female sex	2484 (49.3)	610 (59.3)	522 (52.3)	505 (50.0)	465 (46.1)	382 (38.5)
Observations per child	21.55 ± 0.68	21.40 ± 0.76	21.51 ± 0.69	21.57 ± 0.65	21.63 ± 0.64	21.64 ± 0.63
Linear growth velocity, cm/mo	0.94 ± 0.59	0.85 ± 0.59	0.91 ± 0.58	0.94 ± 0.58	0.98 ± 0.58	1.02 ± 0.58
Age at first measurement, mo	6.16 ± 0.58	6.10 ± 0.59	6.13 ± 0.58	6.13 ± 0.57	6.19 ± 0.58	6.23 ± 0.59
Age at last measurement, mo	26.68 ± 0.75	26.48 ± 0.86	26.61 ± 0.76	26.67 ± 0.74	26.80 ± 0.66	26.86 ± 0.65
Length at first measurement, cm	65.62 ± 2.58	63.06 ± 1.98	64.59 ± 1.71	65.64 ± 1.78	66.55 ± 1.72	68.36 ± 2.06
Length at last measurement, cm	84.96 ± 3.25	80.43 ± 1.66	83.32 ± 0.52	85.01 ± 0.49	86.71 ± 0.51	89.49 ± 1.57
LAZ at first measurement	−0.59 ± 1.06	−1.62 ± 0.87	−1.01 ± 0.71	−0.56 ± 0.77	−0.23 ± 0.69	0.50 ± 0.84
LAZ at last measurement	−1.36 ± 0.97	−2.64 ± 0.59	−1.81 ± 0.34	−1.33 ± 0.32	−0.86 ± 0.30	−0.06 ± 0.51
LAD at first measurement	−1.34 ± 2.37	−3.60 ± 1.95	−2.28 ± 1.62	−1.27 ± 1.75	−0.55 ± 1.60	1.06 ± 1.89
LAD at last measurement	−3.77 ± 3.18	−8.02 ± 1.88	−5.31 ± 1.06	−3.70 ± 1.01	−2.16 ± 0.97	0.47 ± 1.69

^1^Values are means ± SDs or *n* (%). LAD, length-for-age difference; LAZ, length-for-age *z* score.

**FIGURE 1:**
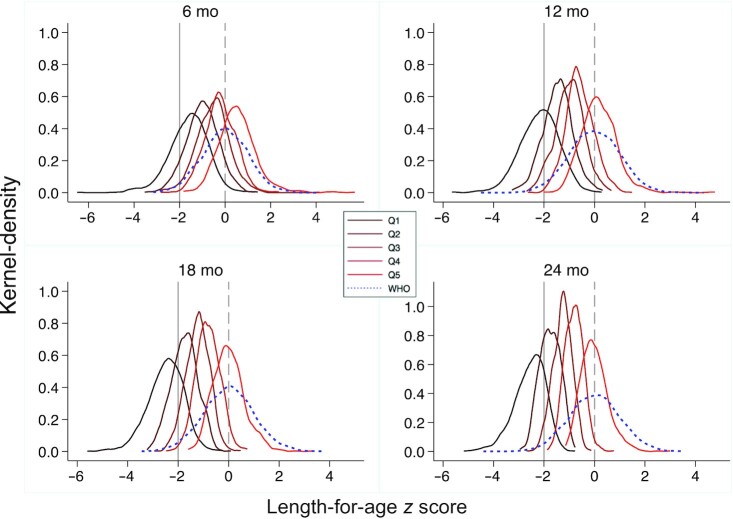
Kernel-density plots of LAZs at ages 6 mo (*n* = 3809), 12 mo (*n* = 4760), 18 mo (*n* = 4776), and 24 mo (*n* = 4807), by quintile of attained length at study end (∼27 mo). The dashed vertical line is the WHO reference population of healthy children whose *z* scores have a mean of 0 and an SD of 1, centered at 0 on the dashed vertical line; the solid vertical line is the stunting cutoff (−2 SD < WHO mean). LAZ, length-for-age *z* score; Q, quintile.

Kernel-density plots of LAZs among the sample children in each quintile compared to the WHO reference at ages 6, 12, 18, and 24 mo showed population-level shifts in LAZ distribution by attained length level (**Figure 1**). Regardless of attained length, the LAZ distribution shifted further to the left of the WHO reference as children aged. By 24 mo, even those with the highest attained length had a LAZ distribution skewed to the left of the WHO reference.

### Visualizations of individual growth curves

**FIGURE 2:**
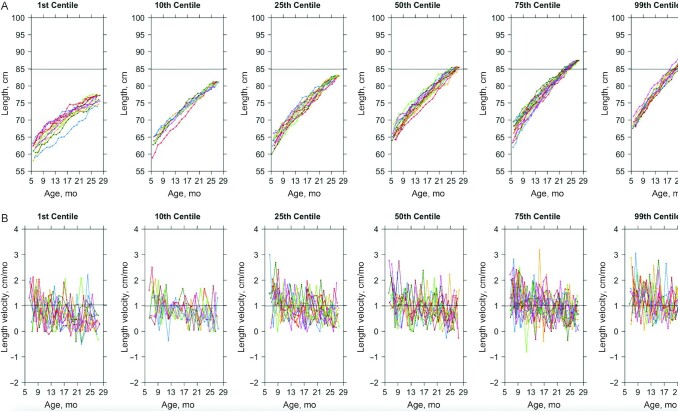
Length (A) and length velocity (B) by age among children from selected centiles of attained length. Each colored line represents the growth curve over time of 1 individual child. Horizontal bars indicate (A) mean attained length and (B) mean length velocity . The graphs use the sensitivity analysis data set containing only full cases with no imputations. *n* = 12–13 in each centile.

**FIGURE 3:**
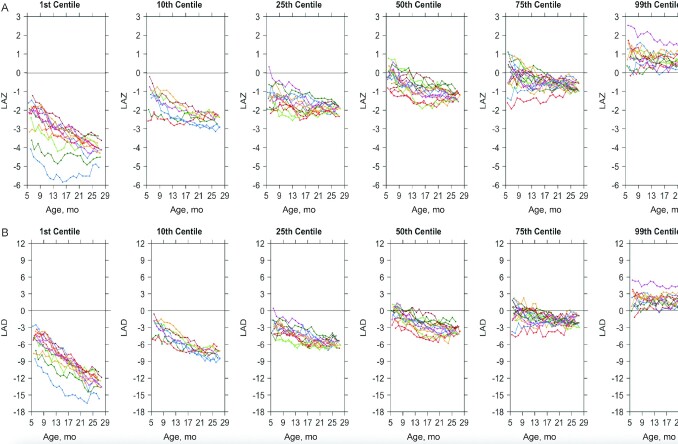
LAZs (A) and LADs (B) by age among children from selected centiles of attained length. Each colored line represents the growth curve over time of 1 individual child. Horizontal bars indicate (A) the reference population *z* score mean and (B) 0 LAD from the reference population median. The graphs use the sensitivity analysis data set containing only full cases with no imputations. *n* = 12–13 in each centile. LAD, length-for-age difference; LAZ, length-for-age *z* score.

We compared individual growth trajectories in selected centiles of attained length (**Figures 2** and **3**). In lower centiles of attained length, growth curves were flatter, and slopes appeared less constant throughout the observed period (Figure 2A). Children with lower attained length started smaller and remained on their initial trajectories, continuously growing slower than taller children. Those in the lowest centile of attained length reached 76 cm by 28 mo, whereas those in the 99^th^ centile reached this length by 10 mo of age. Growth velocity was highly heterogeneous over time among all children, regardless of attained length rank (Figure 2B). Length velocities at each age were similarly normally distributed across quintiles of attained length, implying that heterogeneity in growth velocity was not an artifact of measurement error (**Supplemental Table 4**). Although there was heterogeneity in the rate of growth in all selected centiles, the amplitude of length velocities was smaller among children in the lower centiles.

Examination of LAZ and LAD curves (Figure 3) revealed that children in the lowest centiles of attained length experienced continuous steep declines in growth relative to the reference population as they aged, whereas children in the highest centiles had relatively constant growth rates in relation to the reference population. The steepest declines happened before 12 mo of age; however, LAD curves (Figure 3B) among the lower centiles (1^st^–50^th^) showed that declines continued throughout the study period. Relying only on LAZ, one may conclude that declines in growth, even among the lowest centiles, level out after 12 mo of age; however, LAD is likely a more appropriate indicator to evaluate the extent of linear growth faltering in children as they age (20).

### Growth smoothness and attained length

Two-stage regressions testing the relation between smoothness of growth and attained length revealed that the smoothness of growth curves was associated with higher attained length, but growth velocities were the most influential determinant of attained length. **Table 2** summarizes regression parameters extracted from individual regressions of length on age by attained length quintile. Those with higher attained length had higher intercepts (initial length), *R*^2^ (smoothness of growth), and age coefficients (mean length velocities) from the individual spline regressions. Linear regressions of parameters from individual growth curves on attained length (**Table 3**) revealed that smoothness of growth curves alone was significantly associated with increased length. Without considering growth velocities, each increase of 0.01 in *R*^2^ was associated with a 3.10-cm higher attained length (95% CI: 2.80, 3.41 cm) (Model 1). Sensitivity analyses using alternative functional forms for the individual regression models showed similar results (Supplemental Table 1).

**TABLE 2:** Descriptive statistics of regression diagnostics from individual linear spline regressions of length on age^1^

	Overall (*n* = 5039)	Quintile 1 (*n* = 1028)	Quintile 2 (*n* = 999)	Quintile 3 (*n* = 1010)	Quintile 4 (*n* = 1009)	Quintile 5 (*n* = 993)
Intercept	57.88 ± 3.84	56.07 ± 3.46	57.12 ± 3.17	57.70 ± 3.64	58.52 ± 3.95	60.03 ± 3.71
*R* ^2^	0.9956 ± 0.003	0.9944 ± 0.004	0.9953 ± 0.003	0.9956 ± 0.002	0.9960 ± 0.002	0.9964 ± 0.002
Average velocity, 6–8 mo	1.27 ± 0.40	1.16 ± 0.37	1.24 ± 0.36	1.30 ± 0.39	1.31 ± 0.44	1.35 ± 0.40
Average velocity, 9–11 mo	1.02 ± 0.30	0.95 ± 0.30	1.00 ± 0.29	1.01 ± 0.29	1.06 ± 0.27	1.11 ± 0.30
Average velocity, 12–14 mo	0.94 ± 0.28	0.86 ± 0.27	0.89 ± 0.27	0.94 ± 0.27	0.99 ± 0.26	1.04 ± 0.27
Average velocity, 15–17 mo	0.92 ± 0.27	0.82 ± 0.28	0.89 ± 0.26	0.93 ± 0.27	0.94 ± 0.25	1.01 ± 0.26
Average velocity, 18–20 mo	0.88 ± 0.26	0.77 ± 0.28	0.87 ± 0.25	0.89 ± 0.25	0.92 ± 0.25	0.97 ± 0.24
Average velocity, 21–23 mo	0.82 ± 0.26	0.74 ± 0.28	0.81 ± 0.25	0.81 ± 0.25	0.86 ± 0.24	0.91 ± 0.25
Average velocity, 24–28 mo	0.70 ± 0.51	0.62 ± 0.69	0.65 ± 0.49	0.71 ± 0.44	0.73 ± 0.28	0.79 ± 0.54
*F*-statistic	895.18 ± 539.27	742.23 ± 462.74	841.24 ± 518.16	865.44 ± 510.16	984.45 ± 576.42	1047.33 ± 568.72

^1^Values are means ± SDs. ANOVA tests for significant differences in each variable between quintiles, with Bonferroni correction for multiple comparisons, all showed significant differences (in all cases *P* < 0.0001).

**TABLE 3:** Contribution of uninterrupted growth and age-specific velocities to attained length of children at 28 mo^1^

	Model 1	Model 2	Model 3	Model 4	Model 5	Model 6	Model 7	Model 8	Model 9
*R* ^2^ (smoothness)	3.104*** (2.796, 3.411)	3.370*** (3.113, 3.628)	3.300*** (3.058, 3.543)	2.656*** (2.473, 2.839)	2.092*** (1.938, 2.246)	1.735*** (1.584, 1.886)	1.168*** (1.025, 1.311)	0.567*** (0.442, 0.692)	0.412*** (0.295, 0.529)
Initial length		0.610*** (0.584, 0.636)	0.671*** (0.646, 0.696)	0.946*** (0.926, 0.967)	0.979*** (0.962, 0.996)	0.957*** (0.941, 0.974)	0.943*** (0.928, 0.958)	0.956*** (0.943, 0.969)	0.958*** (0.946, 0.970)
Velocity, 6–8 mo			2.260*** (2.085, 2.435)	3.706*** (3.567, 3.845)	3.677*** (3.561, 3.792)	3.590*** (3.479, 3.701)	3.473*** (3.371, 3.575)	3.441*** (3.354, 3.529)	3.433*** (3.352, 3.514)
Velocity, 9–11 mo				6.296*** (6.098, 6.493)	7.180*** (7.011, 7.348)	7.015*** (6.853, 7.177)	6.841*** (6.693, 6.990)	6.755*** (6.627, 6.882)	6.713*** (6.595, 6.831)
Velocity, 12–14 mo					3.860*** (3.700, 4.020)	4.153*** (3.997, 4.308)	4.062*** (3.919, 4.204)	3.938*** (3.816, 4.060)	3.906*** (3.792, 4.019)
Velocity, 15–17 mo						1.760*** (1.600, 1.920)	2.262*** (2.112, 2.412)	2.213*** (2.084, 2.341)	2.203*** (2.084, 2.322)
Velocity, 18–20 mo							2.383*** (2.234, 2.533)	2.910*** (2.780, 3.041)	2.900*** (2.779, 3.021)
Velocity, 21–23 mo								2.786*** (2.658, 2.914)	3.056*** (2.936, 3.176)
Velocity, 24–28 mo									0.852*** (0.793, 0.911)
Observations	5039	5039	5039	5039	5039	5039	5039	5039	5039
*R* ^2^	0.072	0.350	0.423	0.675	0.775	0.794	0.828	0.873	0.891

^1^Values are centimeters (95% CIs) of attained length associated with each increase of 0.01 in *R*^2^, each 1-cm increase in initial length, and each 1-cm increase in length gained per month during the specified age block, unless otherwise indicated. Coefficients displayed are results of 2-stage regressions; stage 1 was regression of each individual child's length on their age using linear spline regressions with 6 knots; stage 2 was regression of extracted model parameters from stage 1 on attained length at 27 mo, using ordinary least squares regressions. Sensitivity analyses using alternative functional forms for the individual regression models (linear polynomial model with cubic term for age, restricted cubic splines, linear splines with 4 knots) gave a range of 2.44–2.83 for *R*^2^ when attained length was regressed on *R*^2^, with 95% CIs spanning 2.39–3.09. ***P < 0.001.

As additional parameters from individual linear spline models were added into the model for attained length, the importance of *R*^2^ decreased in relation to mean growth velocities but remained statistically significant (Models 2–9). This is expected, because the only roughness of growth to explain once all growth velocity variables have been included occurs within the 3-mo spline intervals. The most influential age period for growth was between 9 and 11 mo; during this period, for each 1-cm increase in length gained per month, children achieved an additional 6.71 cm of length at the end of the study period, compared with 3.91 cm in the next most influential period from 12 to 14 mo.

### Changes in monthly growth rates and attained length

Analysis of how variation of changes in monthly growth rates is related to attained length at 27 mo of age showed that slow growth periods, defined as 2 consecutive months of growth <15^th^ centile compared with others in the sample, were related to shorter attained lengths in children (**Tables 4** and **5**). A much larger proportion of children with the lowest attained length had ≥1 instance of slow growth (40.7%) than among those with the highest attained length (14.8%). Episodes of slow growth were also more frequent among shorter children (3.9 episodes) than among taller children (2.4 episodes) and lasted longer (1.5 mo and 1.1 mo, respectively) (Table 4).

**TABLE 4:** Frequency and duration of episodes of slow growth among children throughout the study period^1^

	Overall		Q1		Q2		Q3		Q4		Q5	
Definition of slow growth	Overall (*n* = 103,541)	Child (*n* = 5039)	Overall (*n* = 20,968)	Child (*n* = 1028)	Overall (*n* = 20,490)	Child (*n* = 999)	Overall (*n* = 20,774)	Child (*n* = 1010)	Overall (*n* = 20,816)	Child (*n* = 1009)	Overall (*n* = 20,493)	Child (*n* = 993)
Frequency of slow growth periods												
Months withlength velocity<15^th^ centilefor age	15,581 (15.1)	4844 (96.1)	4041 (19.2)	1014 (98.6)	3318 (16.2)	979 (98.0)	3123 (15.0)	978 (96.8)	2734 (13.1)	961 (95.2)	2365 (11.5)	912 (91.8)
Two consecutivemonths with lengthvelocity <15^th^centile for age	1643 (1.6)	1283 (25.5)	593 (2.8)	418 (40.7)	370 (1.81)	304 (30.4)	302 (1.5)	229 (22.7)	208 (1.0)	185 (18.3)	170 (0.8)	147 (14.8)
Mean number of slow growth period months per child												
Months withlength velocity<15^th^ centilefor age, *n*		3.1 ± 1.65		3.94 ± 1.76		3.33 ± 1.55		3.10 ± 1.59		2.72 ± 1.47		2.39 ± 1.42
Longest duration of slow growth periods												
Months withlength velocity<15^th^ centilefor age		1.28 ± 0.65		1.51 ± 0.72		1.35 ± 0.60		1.25 ± 0.78		1.17 ± 0.52		1.10 ± 0.54

^1^Values are means ± SDs or *n* (%). Chi-square and ANOVA tests for significant differences in each variable between quintiles, with Bonferroni correction for multiple comparisons, all showed significant differences (in all cases *P* < 0.0001). Q, quintile of attained length.

**TABLE 5:** Relation between attained length (cm) and periods of slow growth^1^

Variables	Slow growth for 2 mo	Slow growth frequency	Slow growth duration
Length velocity < 15^th^ centile at 2 consecutive time points	−1.696*** (−1.802, −1.590)		
Instances of growth < 15^th^ centile		−0.694*** (−0.700, −0.687)	
Longest duration of consecutive measurements < 15^th^ centile			−1.253*** (−1.271, −1.234)
Study arm (Ref = CSB+)			
CSWB	−0.828*** (−0.866, −0.789)	−0.550*** (−0.583, −0.518)	−0.707*** (−0.742, −0.672)
SC+	0.074*** (0.037, 0.111)	0.054** (0.023, 0.085)	0.061*** (0.027, 0.095)
RUSF	−0.190*** (−0.228, −0.151)	−0.069*** (−0.101, −0.036)	−0.172*** (−0.207, −0.137)
Total illness episodes over study period, *n*	−0.104*** (−0.113, −0.096)	−0.042*** (−0.049, −0.035)	−0.079*** (−0.087, −0.072)
Length at first measurement, cm	0.938*** (0.933, 0.943)	0.940*** (0.936, 0.944)	0.946*** (0.941, 0.951)
Observations	103,541	108,580	108,580
*R* ^2^	0.551	0.669	0.610

^1^Values are results of linear regressions (ordinary least squares) and are centimeters of attained length associated with periods of slow growth, as defined by having 2 consecutive length velocity (cm/mo) measurements < 15^th^ centile as compared with the sample population, frequency of measurements < 15^th^ centile, and longest duration of consecutive measurements < 15^th^ centile; 95% CIs are in parentheses. ***P < 0.001, **P < 0.01. Total number of illness episodes over study period self-reported by caregivers at each measurement visit. CSB+, corn–soy blend plus; CSWB, corn–soy–whey blend; RUSF, ready-to-use supplementary food; SC+, SuperCereal plus.

On average, after controlling for study arm, length at first measurement, and total number of illness episodes over the study period, 2 consecutive length velocity measurements <15^th^ centile as compared with the sample population was associated with 1.7-cm lower attained lengths (95% CI: −1.80, −1.59 cm) than for those who did not experience periods of slow growth by this definition. With every unit increase in frequency of episodes where growth was <15^th^ centile, attained length was 0.69 cm lower (95% CI: −0.70, −0.68 cm), and with every unit increase in the longest duration of slow growth episodes, attained length was 1.25 cm lower (95% CI: −1.27, −1.23 cm) (Table 5). Sensitivity analyses using a threshold for slow growth of 2 consecutive negative residuals from growth models, which account for the autocorrelative nature of the growth process, showed the same direction of effect, at a smaller magnitude, owing to the high frequency of such events in the data set (**Supplemental Table 5**).

Age at slow growth onset was itself an important predictor of attained length; if a slow growth period first occurred when children were 7–8 mo old (the earliest possible observation among this sample), children were on average 2.4 cm (95% CI: −2.49, −2.27 cm) shorter at the end of the study period than if they never experienced any periods of slow growth. Later onset of slow growth periods had less of an effect on attained length, although still a significant one—the smallest effect was for onset between 24 and 28 mo, which was still associated with attained lengths 1.37 cm lower than for those with no periods of slow growth (**Supplemental Table 6**).

## Discussion

Using multiple methods to examine individual growth curves among young children in Burkina Faso, we have found that the shortest children started small and stayed on their initial growth trajectories, continuously growing slower than those who were in the highest quintile of the population distribution of attained length at 27 mo. We demonstrate the importance of smooth growth for attained length, including the detrimental effects of punctual episodes of slower growth, as well as the relative influence of growth velocities at different age periods. At 6–27 mo of age, growth faltering manifests through consistently slow growth, as well as greater levels of heterogeneity in growth velocities with frequent episodes of slower growth.

That children who end shorter start with slower growth velocities that continue to decline as they age confirms that the timing of growth faltering among individuals closely resembles what has been concluded based on cross-sectional studies of population averages. Such studies have been consistent in showing that children are often born with LAZs already <0, and that children have lower mean LAZ at each increasing age (1, 3, 8, 21). Longitudinal studies of child growth in Malawi and the Gambia, as well as an analysis of pooled data from 31 longitudinal cohorts, have found similarly that linear growth faltering starts at birth and continues throughout the first 3 y of life, with larger deficits at younger ages informing higher incidence of later stunting (10, 22, 23). Findings from our analyses of individual growth curves confirm that children who start with larger deficits in relation to the WHO standards maintain and increase these deficits. Thus, the addition of children to the stunted category of LAZ < −2 as they age is a function of children who already had lower LAZs continuing to lose LAZ as they age.

In our study population, as has been seen in other studies set in low- and middle-income countries (1, 3, 24), despite a nutrition intervention covering the entire region, the distribution of LAZs was shifted to the left of the WHO growth reference distribution, and got further to the left as the children age. Clearly, population-wide conditions are not conducive to optimal child growth and development (3, 25). Even so, we found significant variation in trajectories, including evidence that growth tempo plays a key role in overall height attainment. Children in the lowest centile of attained length reached ∼76 cm by 28 mo of age, whereas those in the 99^th^ centile had reached this length by 10 mo. Slower growth tempos, contributing to larger growth delays, may also influence attained height (26). Further, the comparison of LAD with LAZ in our sample, in which we found the largest differences between LAD and LAZ to be among the shortest children, corroborates important conclusions by Leroy et al. (9, 20) regarding limitations of the *z* score approach, which does not accurately reflect the continued linear growth deficits that children experience in low-resource settings. Using indicators other than LAZ to assess growth over time in children has consistently shown that, for the most vulnerable children, limiting interventions to children <2 y old may be insufficient to prevent further growth faltering.

We show, to our knowledge for the first time, that the smoothness of a child's growth is an important factor in relation to attained length. Children who were shorter at 2 y of age not only had slower velocity in each month but also greater variation in velocity, including more frequent and longer episodes of slow growth than taller children. This suggests that consecutive months of slow growth may be an early warning of lower future growth and indicates a significant role for episodic growth insults at multiple time points. Although we lack data on growth between birth and 6 mo, a period which has previously been shown to contain the highest stunting incidence (between 0 and 3 mo) (10), we demonstrate that there are additional influential growth windows to consider when designing interventions.

Effects of increased growth heterogeneity and episodes of slow growth are more pronounced if they occur among younger children, but are still significant if onset happens at older ages. In addition, growth between 9 and 11 mo may be especially influential, because length velocity during this period is associated with almost twice the increases in attained length compared with the next most influential period (12–14 mo). This period from 9 to 11 mo may be related to the transition of the child from exclusive breastfeeding to complementary feeding; although children often begin this transition at the recommended age of 6 mo (8, 27), use of household complementary foods is minimal until ∼9 mo, when the WHO recommends increasing meal frequency and quantity, which are often far from adequate (28, 29), In contexts such as Burkina Faso, transition to complementary household foods may pose challenges to child growth owing to increased exposure to pathogens and food insecurity that limits the quality and safety of complementary foods (28, 30).

If conditions are favorable to growth, with adequate micro- and macronutrient intake for the biological processes that regulate bone growth and limited environmental exposure to infection and inflammation, a child will grow to their genetic potential (31). Episodic growth insults, in addition to the chronically slow growth caused by earlier or continuous exposure to adverse factors that constrain optimal child growth, make achieving this genetic potential for height challenging in contexts such as Burkina Faso. Catch-up growth may be possible in some contexts, whereby growth velocity exceeds the normal statistical range for a child's age for a period of time, to bring them back to their original growth trajectory after episodic growth insults (32). However, such catch-up is unlikely to occur when children chronically live in conditions that do not favor optimal growth and in which children are repeatedly exposed to growth insults (31–33).

We note several limitations to our study design. First, the lack of data on maternal height, pregnancy histories, birth size, and measurements ≤6 mo of age, given the importance of fetal growth restriction and size at birth (10, 31, 34–36). We are thus unable to condition our results on genetic factors that may influence growth curves and cannot account for heterogeneity in gestational age at birth, which can affect growth in the first 2 y of life. We assume the initial length measurements in our study to be an indicator of the cumulative growth velocities in the first 6 mo of life. In addition, because these are secondary analyses, all children were part of the supplementary feeding intervention trial, so we cannot determine how growth faltering would have happened in the absence of the intervention program. Nonetheless, growth faltering patterns were consistent across supplementation groups. Lastly, the data were collected from a sample of children in 1 province in Burkina Faso and may not be representative of children in every low- or middle-income country setting. However, the results align well with many other studies from low- and middle-income countries, suggesting some level of generalizability.

Our use of longitudinal data that follows a single cohort of children each month from ∼6 to 28 mo adds important nuances to discussions of the timing of growth faltering that have implications for the optimal timing and nature of growth interventions. Findings point to the utility of addressing the overall conditions in which children live that constrain their growth and underline the importance of improving community-level systemic factors that constrain growth among the entire population, rather than simply focusing on household or nutritional factors. In addition, growth surveillance programs could plausibly detect early signs of growth faltering using a minimum of 3 consecutive measurements (to get ≥2 consecutive growth velocity measurements) if the measurements are taken at sufficiently high frequency to avoid missingness. Further research should use anthropometric indexes appropriate for the study of longitudinal growth (i.e., LAD, length velocity, etc.) to examine the factors that contribute to slow growth periods, validate the threshold for slow growth episodes, explore both relative and absolute thresholds, and determine the number of months of consecutively slower growth that would balance the sensitivity and specificity of identifying growth faltering early in growth monitoring programs.

## Supplementary Material

nqab309_Supplemental_File

## Data Availability

De-identified anthropometric data used in this study are available at https://data.usaid.gov. The study protocol and analytic code are available upon request.

## Notes

Supported by United States Agency for International Development (USAID) grant AID-OAA-C-16-00020 (to BLR), through the Food Aid Quality Review project. The funder had no part in this study.

Supplemental Methods 1, Supplemental Figure 1, and Supplemental Tables 1–6 are available from the “Supplementary data” link in the online posting of the article and from the same link in the online table of contents at https://academic.oup.com/ajcn/.

Abbreviations used: IRB, Institutional Review Board; LAD, length-for-age difference; LAZ, length-for-age *z* score.
