# Supplementary material for: Monthly measurement of child lengths between 6 and 27 months of age in Burkina Faso reveals both chronic and episodic growth faltering
Source: Am J Clin Nutr. 2021 Oct 12;115(1):94–104. doi: 10.1093/ajcn/nqab309 (PMC8755055; doi:10.1093/ajcn/nqab309)
Supplement: nqab309_Supplemental_File [file nqab309_supplemental_file.docx]

# **On-line Supplementary Material**: Cliffer et al., Monthly measurement of child lengths between 6-27 months in Burkina Faso reveals both chronic and episodic growth faltering

**Supplementary Methods 1: Data preparation**

Biologically implausible anthropometric values were classified using jackknifed residuals, according to the methods described in Shi et al. 2018 for identification of outliers in longitudinal growth data (1). Jackknifed residuals from regression of length and weight measurements on the square root of age with absolute values above 5 were flagged as biologically implausible and excluded. Roughly 7% of measurements from the original dataset (8,444 observations) were identified as biologically implausible or missing, and imputed using linear predictions from ordinary least-squares regression of length on age, with each child missing one value on average. Missingness and implausibility were not found to be related to enumerators or socio-demographic variables.

Though we aimed to enroll children in our study at ~6 months of age and follow each child for 21 months (18 months during the intervention + 3 months post-intervention) until they reached 27 months of age, children in our sample range from 1-37 months, with the vast majority (99%) falling between 5-28 months. We thus restrict our analyses to children 5-28 months, though most analyses are done on children 6-27 months as the sample sizes are small among those at the extremes. As our primary outcome is attained length at the end of the study period, we further restrict analyses to children who had at least 20 repeated measurements.

**References**

1. Shi J, Korsiak J, Roth DE. New approach for the identification of implausible values and outliers in longitudinal childhood anthropometric data. Annals of Epidemiology. 2018;28(3):204-211.e3. https://doi.org/10.1016/j.annepidem.2018.01.007. doi:10.1016/j.annepidem.2018.01.007

**Supplementary Figures**

**
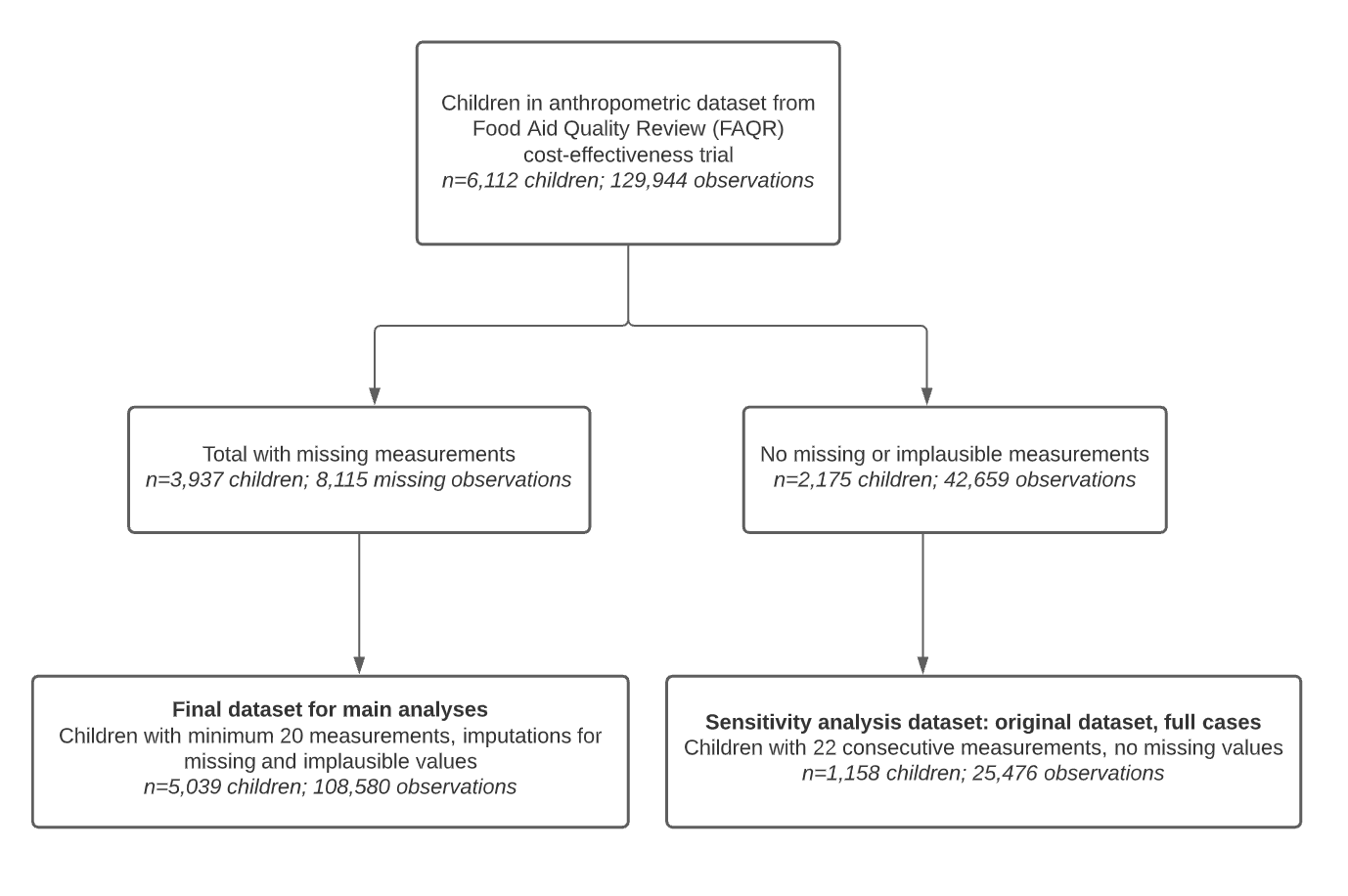
**

**Supplementary Figure 1.** Participant flowchart for children included in secondary analysis of Food Aid Quality Review (FAQR) anthropometric data

**Supplementary Tables**

**Supplementary Table 1. Sample anthropometric characteristics by quintile of attained length, sensitivity analysis dataset**

|  | Sensitivity analysis dataset | | | | | | | |  |
| --- | --- | --- | --- | --- | --- | --- | --- | --- | --- |
|  | Overall (N=1,158) | Quintile 1 (n=236) | Quintile 2 (n=228) | Quintile 3 (n=231) | | Quintile 4 (n=232) | | Quintile 5 (n=231) | |
| Total # observations | 25,476 | 5,192 | 5,016 | 5,082 | | 5,104 | | 5,082 | |
| Female sex | 532 (45·9) | 138 (58·5) | 115 (50·4) | 106 (45·9) | | 96 (41·4) | | 77 (33·3) | |
| Observations per child | 22·0 ± 0·00 | 22·0 ± 0·00 | 22·0 ± 0·00 | 22·0 ± 0·00 | | 22·0 ± 0·00 | | 22·0 ± 0·00 | |
| Linear growth velocity (cm/month) | 0·94 ± 0·56 | 0·84 ± 0·56 | 0·91 ± 0·55 | 0·94 ± 0·56 | | 0·97 ± 0·55 | | 1·02 ± 0·57 | |
| Age at first measurement, months | 6·00 ± 0·38 | 5·94 ± 0·38 | 5·96 ± 0·36 | 5·99 ± 0·42 | | 6·05 ± 0·36 | | 6·07 ± 0·35 | |
| Age at last measurement, months | 27·00 ± 0·40 | 26·90 ± 0·41 | 26·94 ± 0·43 | 26·95 ± 0·43 | | 27·02 ± 0·36 | | 27·04 ± 0·37 | |
| Length (cm) at first measurement | 65·52 ± 2·50 | 62·98 ± 2·06 | 64·38 ± 1·68 | 65·49 ± 1·54 | | 66·58 ± 1·67 | | 68·18 ± 1·78 | |
| Length (cm) at last measurement | 85·23 ± 3·26 | 80·68 ± 1·62 | 83·47 ± 0·54 | 85·36 ± 0·55 | | 86·98 ± 0·49 | | 89·71 ± 1·51 | |
| Length-for-age z-score at first measurement | -0·59 ± 1·03 | -1·56 ± 0·93 | -1·01 ± 0·73 | -0·57 ± 0·67 | | -0·16 ± 0·64 | | 0·51 ± 0·77 | |
| Length-for-age z-score at last measurement | -1·35 ± 0·97 | -2·65 ± 0·57 | -1·86 ± 0·29 | -1·31 ± 0·32 | | -0·85 ± 0·27 | | -0·06 ± 0·49 | |
| Length-for-age difference at first measurement | -1·27 ± 2·31 | -3·47 ± 2·06 | -2·26 ± 1·62 | -1·28 ± 1·53 | | -0·37 ± 1·53 | | 1·05 ± 1·72 | |
| Length-for-age difference at last measurement | -3·77 ± 3·20 | -8·12 ± 1·79 | -5·44 ± 0·89 | -3·63 ± 1·01 | | -2·13 ± 0·87 | | 0·51 ± 1·59 | |
| Notes: Values are mean ± SD or n (%) | | | | |  | |  | |  |

| **Supplementary Table 2. Sensitivity analyses by functional form for age** | | | | |
| --- | --- | --- | --- | --- |
|  | Cubic Polynomial | 4-Knot Linear Splines | 6-Knot Linear Splines | Cubic Splines |
| R^2^ (smoothness) | 2·442*** | 2·532*** | 3·108*** | 2·841*** |
|  | *(2·394, 2·491)* | *(2·480, 2·584)* | *(3·042, 3·174)* | *(2·787, 2·896)* |
| Observations | 107,677 | 108,802 | 108,802 | 108,802 |
| R-squared | 0·082 | 0·076 | 0·073 | 0·086 |

Note: Values are regression coefficient results showing cm of attained length associated with each increase of 0.01 in R^2^ from candidate models testing different functional forms for the first stage of two-stage linear regression models. Stage 1 was regression of each individual child’s length on their age, using one of the four candidate functional forms displayed. Stage 2 was ordinary least squares regression of extracted model parameters from stage 1 on attained length.

95% confidence interval in parentheses

*** p<0·01, ** p<0·05, * p<0·1

| **Supplementary Table 3. Mean R^2^ by quintile in each study arm** | | | | | |
| --- | --- | --- | --- | --- | --- |
|  | Quintile 1 | Quintile 2 | Quintile 3 | Quintile 4 | Quintile 5 |
| CSB+ | 0·9946 | 0·9955 | 0·9958 | 0·9964 | 0·9964 |
| CSWB | 0·9939 | 0·9953 | 0·9955 | 0·9960 | 0·9962 |
| SC+ | 0·9948 | 0·9956 | 0·9958 | 0·9961 | 0·9965 |
| RUSF | 0·9945 | 0·9951 | 0·9952 | 0·9958 | 0·9963 |

Note: Values are mean R^2^ in each study arm from stage 1 of two-stage linear regression models. Stage 1 was regression of each individual child’s length on their age, using one of the four candidate functional forms displayed. Stage 2 was ordinary least squares regression of extracted model parameters from stage 1 on attained length.

| **Supplementary Table 4. Length velocity moments by age in each quintile** | | | | | | | | | | | | | | | | | | | | |
| --- | --- | --- | --- | --- | --- | --- | --- | --- | --- | --- | --- | --- | --- | --- | --- | --- | --- | --- | --- | --- |
|  | **Quintile 1** | | | | **Quintile 2** | | | | **Quintile 3** | | | | **Quintile 4** | | | **Quintile 5** | | | | |
| **Age** | **N** | **LV** | **SD** | **CV** | **N** | **LV** | **SD** | **CV** | **N** | **LV** | **SD** | **CV** | **N** | **LV** | **SD** | **CV** | **N** | **LV** | **SD** | **CV** |
| 6 | 130 | 1·28 | 0·76 | 0·59 | 88 | 1·63 | 0·69 | 0·42 | 103 | 1·56 | 0·75 | 0·48 | 63 | 1·58 | 0·81 | 0·51 | 59 | 1·58 | 0·77 | 0·49 |
| 7 | 788 | 1·29 | 0·75 | 0·58 | 771 | 1·39 | 0·72 | 0·52 | 746 | 1·45 | 0·73 | 0·50 | 728 | 1·50 | 0·74 | 0·49 | 709 | 1·48 | 0·74 | 0·50 |
| 8 | 998 | 1·12 | 0·66 | 0·59 | 976 | 1·20 | 0·66 | 0·55 | 999 | 1·25 | 0·68 | 0·54 | 990 | 1·27 | 0·66 | 0·52 | 936 | 1·36 | 0·66 | 0·49 |
| 9 | 1,009 | 1·06 | 0·61 | 0·58 | 986 | 1·12 | 0·61 | 0·54 | 1,007 | 1·15 | 0·63 | 0·55 | 980 | 1·21 | 0·62 | 0·51 | 978 | 1·25 | 0·64 | 0·51 |
| 10 | 1,008 | 1·01 | 0·59 | 0·58 | 965 | 1·04 | 0·57 | 0·55 | 974 | 1·11 | 0·59 | 0·53 | 991 | 1·09 | 0·56 | 0·51 | 967 | 1·14 | 0·57 | 0·50 |
| 11 | 1,026 | 0·97 | 0·59 | 0·61 | 1,003 | 1·02 | 0·57 | 0·56 | 1,021 | 1·01 | 0·55 | 0·54 | 1,033 | 1·08 | 0·56 | 0·52 | 1,006 | 1·12 | 0·57 | 0·51 |
| 12 | 1,026 | 0·89 | 0·57 | 0·64 | 990 | 0·98 | 0·60 | 0·61 | 982 | 0·97 | 0·55 | 0·57 | 973 | 1·05 | 0·59 | 0·56 | 992 | 1·11 | 0·57 | 0·51 |
| 13 | 1,020 | 0·90 | 0·56 | 0·62 | 1,002 | 0·93 | 0·56 | 0·60 | 1,025 | 0·95 | 0·57 | 0·60 | 1,014 | 1·01 | 0·59 | 0·58 | 982 | 1·04 | 0·57 | 0·55 |
| 14 | 1,017 | 0·85 | 0·56 | 0·66 | 984 | 0·87 | 0·57 | 0·66 | 997 | 0·95 | 0·61 | 0·64 | 1,004 | 0·97 | 0·57 | 0·59 | 983 | 1·04 | 0·56 | 0·54 |
| 15 | 1,022 | 0·86 | 0·55 | 0·64 | 983 | 0·90 | 0·54 | 0·60 | 981 | 0·94 | 0·55 | 0·59 | 995 | 0·99 | 0·55 | 0·56 | 966 | 1·01 | 0·59 | 0·58 |
| 16 | 1,036 | 0·82 | 0·56 | 0·68 | 1,024 | 0·88 | 0·55 | 0·63 | 1,029 | 0·92 | 0·54 | 0·59 | 997 | 0·98 | 0·59 | 0·60 | 1,015 | 1·05 | 0·58 | 0·55 |
| 17 | 1,007 | 0·85 | 0·51 | 0·60 | 978 | 0·91 | 0·52 | 0·57 | 1,001 | 0·94 | 0·55 | 0·59 | 1,008 | 0·96 | 0·56 | 0·58 | 974 | 1·02 | 0·58 | 0·57 |
| 18 | 1,047 | 0·81 | 0·56 | 0·69 | 1,005 | 0·90 | 0·54 | 0·60 | 1,020 | 0·92 | 0·56 | 0·61 | 1,033 | 0·92 | 0·54 | 0·59 | 1,008 | 1·00 | 0·56 | 0·56 |
| 19 | 1,020 | 0·79 | 0·58 | 0·73 | 1,000 | 0·87 | 0·54 | 0·62 | 1,001 | 0·93 | 0·54 | 0·58 | 977 | 0·95 | 0·53 | 0·56 | 981 | 0·95 | 0·51 | 0·54 |
| 20 | 1,051 | 0·77 | 0·51 | 0·66 | 1,033 | 0·86 | 0·55 | 0·64 | 1,048 | 0·88 | 0·53 | 0·60 | 1,064 | 0·93 | 0·52 | 0·56 | 1,035 | 0·96 | 0·54 | 0·56 |
| 21 | 1,031 | 0·77 | 0·52 | 0·68 | 995 | 0·84 | 0·51 | 0·61 | 987 | 0·87 | 0·53 | 0·61 | 933 | 0·90 | 0·50 | 0·56 | 984 | 1·00 | 0·52 | 0·52 |
| 22 | 1,028 | 0·74 | 0·50 | 0·68 | 997 | 0·85 | 0·52 | 0·61 | 1,020 | 0·84 | 0·50 | 0·60 | 1,011 | 0·90 | 0·50 | 0·56 | 988 | 0·92 | 0·49 | 0·53 |
| 23 | 1,042 | 0·76 | 0·52 | 0·68 | 1,034 | 0·81 | 0·48 | 0·59 | 1,042 | 0·81 | 0·49 | 0·60 | 1,041 | 0·85 | 0·48 | 0·56 | 1,026 | 0·89 | 0·47 | 0·53 |
| 24 | 1,036 | 0·69 | 0·49 | 0·71 | 995 | 0·73 | 0·46 | 0·63 | 998 | 0·75 | 0·49 | 0·65 | 1,004 | 0·83 | 0·47 | 0·57 | 980 | 0·89 | 0·47 | 0·53 |
| 25 | 1,042 | 0·66 | 0·54 | 0·82 | 1,017 | 0·73 | 0·47 | 0·64 | 1,032 | 0·75 | 0·50 | 0·67 | 1,024 | 0·75 | 0·46 | 0·61 | 1,006 | 0·80 | 0·47 | 0·59 |
| 26 | 898 | 0·64 | 0·52 | 0·81 | 923 | 0·68 | 0·52 | 0·76 | 943 | 0·69 | 0·48 | 0·70 | 977 | 0·75 | 0·50 | 0·67 | 953 | 0·78 | 0·48 | 0·62 |
| 27 | 674 | 0·52 | 0·59 | 1·13 | 706 | 0·61 | 0·52 | 0·85 | 754 | 0·71 | 0·50 | 0·70 | 830 | 0·72 | 0·50 | 0·69 | 852 | 0·75 | 0·51 | 0·68 |
| 28 | 79 | 0·53 | 0·55 | 1·04 | 83 | 0·50 | 0·63 | 1·26 | 106 | 0·64 | 0·57 | 0·89 | 118 | 0·67 | 0·48 | 0·72 | 146 | 0·73 | 0·60 | 0·82 |
| Note: LV = mean length velocity (cm/month); SD = standard deviation; CV = coefficient of variation | | | | | | | | | | | | | | | | | | | | |

| **Supplementary Table 5. Relationship between slow growth and attained length (cm), sensitivity analyses with negative residuals** | | | |
| --- | --- | --- | --- |
| VARIABLES | Slow growth | Slow growth frequency | Slow growth duration |
|  |  |  |  |
| Slow growth – 2 consecutive negative residuals from growth model | -0.149*** |  |  |
|  | (-0.176, -0.122) |  |  |
| Instances of consecutive negative residuals |  | -0.155*** |  |
|  |  | (-0.162, -0.149) |  |
| Longest duration of slow growth |  |  | -0.105*** |
|  |  |  | (-0.111, -0.010) |
|  |  |  |  |
| Study arm (Ref = CSB+) |  |  |  |
| CSWB | -0.834*** | -0.809*** | -0.834*** |
|  | (-0.872, -0.797) | (-0.846, -0.772) | (-0.872, -0.797) |
| SC+ | 0.077*** | 0.081*** | 0.077*** |
|  | (0.040, 0.114) | (0.044, 0.117) | (0.040, 0.113) |
| RUSF | -0.190*** | -0.201*** | -0.190*** |
|  | (-0.228, -0.152) | (-0.238, -0.163) | (-0.228, -0.152) |
| Total # illness episodes over study period | -0.106*** | -0.098*** | -0.105*** |
|  | (-0.114, -0.098) | (-0.106, -0.090) | (-0.114, -0.097) |
| Length at first measurement (cm) | 0.938*** | 0.940*** | 0.938*** |
|  | (0.932, 0.943) | (0.935, 0.946) | (0.933, 0.943) |
|  |  |  |  |
| Observations | 108,580 | 108,580 | 108,580 |
| R-squared | 0.548 | 0.556 | 0.548 |

Note: Values are results of ordinary least squares regressions and are cm of attained length associated with periods of slow growth, as defined by having two consecutive negative residuals (conditional measures) from regressions of length on age to obtain the portion of length at each age uncorrelated with length at the previous age, frequency of slow growth defined in the same way, and longest duration of slow growth defined in the same way; (95% confidence interval); *** p<0.001, ** p<0.01, * p<0.05. CSB+ = Corn soy blend plus, CSWB = Corn soy whey blend, SC+ = SuperCereal plus, RUSF = Ready-to-use-supplementary food. Total # illness episodes over study period self-reported by caregivers at each measurement visit.

| **Supplementary Table 6. Relationship between age at slow growth onset and attained length (cm)** | | | |
| --- | --- | --- | --- |
| VARIABLES | | Slow growth onset | |
|  | |  | |
| Age at slow growth onset (Ref = no slow growth periods) | |  | |
| 7-8 months | | -2.382*** | |
|  | | (-2.490, -2.274) | |
| 9-11 months | | -1.495*** | |
|  | | (-1.557, -1.432) | |
| 12-14 months | | -1.674*** | |
|  | | (-1.736, -1.612) | |
| 15-17 months | | -1.459*** | |
|  | | (-1.523, -1.395) | |
| 18-20 months | | -1.918*** | |
|  | | (-1.984, -1.852) | |
| 21-23 months | | -1.627*** | |
|  | | (-1.695, -1.559) | |
| 24-28 months | | -1.371*** | |
|  | | (-1.428, -1.314) | |
| Study arm (Ref = CSB+) | |  | |
| CSWB | | -0.715*** | |
|  | | (-0.751, -0.680) | |
| SC+ | | 0.056*** | |
|  | | (0.021, 0.090) | |
| RUSF | | -0.161*** | |
|  | | (-0.197, -0.126) | |
| Total # illness episodes over study period | | -0.083*** | |
|  | | (-0.091, -0.075) | |
| Length at first measurement (cm) | | 0.937*** | |
|  | | (0.932, 0.942) | |
|  | |  | |
| Observations | | 108,580 | |
| R-squared | | 0.595 | |

Note: Values are results of ordinary least squares regressions and are cm of attained length associated with age at onset of slow growth periods, as defined by having two consecutive length velocity (cm/month) measurements below the 15^th^ centile as compared to the sample population; (95% confidence interval); *** p<0.001, ** p<0.01, * p<0.05. CSB+ = Corn soy blend +, CSWB = Corn soy whey blend, SC+ = SuperCereal plus, RUSF = Ready-to-use-supplementary food. Total # illness episodes over study period self-reported by caregivers at each measurement visit.
